# Epithelial stem cell homeostasis in Meibomian gland development, dysfunction, and dry eye disease

**DOI:** 10.1172/jci.insight.151078

**Published:** 2021-10-22

**Authors:** Edem Tchegnon, Chung-Ping Liao, Elnaz Ghotbi, Tracey Shipman, Yong Wang, Renee M. McKay, Lu Q. Le

**Affiliations:** 1 Department of Dermatology and; 2 Genetics, Development and Disease Graduate Program, University of Texas Southwestern Medical Center, Dallas, Texas, USA.; 3 Graduate Institute of Medical Sciences, College of Medicine, Taipei Medical University, Taipei, Taiwan.; 4 Hamon Center for Regenerative Science and Medicine,; 5 Simmons Comprehensive Cancer Center, and; 6 O’Donnell Brain Institute, University of Texas Southwestern Medical Center, Dallas, Texas, USA.

**Keywords:** Ophthalmology, Adult stem cells, Mouse models

## Abstract

Dry eye disease affects over 16 million adults in the US, and the majority of cases are due to Meibomian gland dysfunction. Unfortunately, the identity of the stem cells involved in Meibomian gland development and homeostasis is not well elucidated. Here, we report that loss of *Krox20*, a zinc finger transcription factor involved in the development of ectoderm-derived tissues, or deletion of KROX20-expressing epithelial cells disrupted Meibomian gland formation and homeostasis, leading to dry eye disease secondary to Meibomian gland dysfunction. Ablation of *Krox20*-lineage cells in adult mice also resulted in dry eye disease, implicating *Krox20* in homeostasis of the mature Meibomian gland. Lineage-tracing and expression analyses revealed a restricted KROX20 expression pattern in the ductal areas of the Meibomian gland, although *Krox20*-lineage cells generate the full, mature Meibomian gland. This suggests that KROX20 marks a stem/progenitor cell population that differentiates to generate the entire Meibomian gland. Our *Krox20* mouse models provide a powerful system that delineated the identity of stem cells required for Meibomian gland development and homeostasis and can be used to investigate the factors underlying these processes. They are also robust models of Meibomian gland dysfunction–related dry eye disease, with a potential for use in preclinical therapeutic screening.

## Graphical abstract

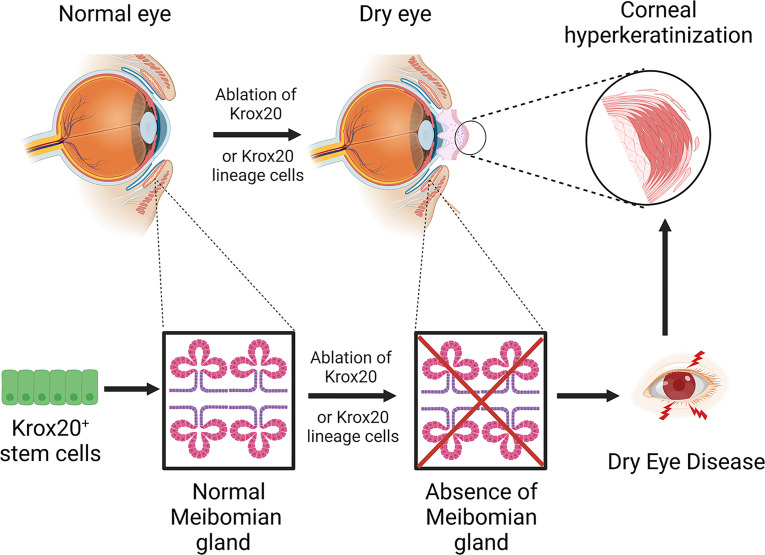

## Introduction

Dry eye disease is one of the most common ocular surface issues, affecting over 16 million adults in the US, and its prevalence continues to increase (1). Dry eye disease occurs when there is inadequate lubrication of the eyes due to insufficient tears or excessive tear evaporation. Symptoms of dry eye disease include burning, stinging, itchy, and red eyes. If left untreated, dry eye disease can lead to more serious problems, including inflammation of the eye, corneal abrasions, corneal ulcers, and corneal hyperkeratinization that can lead to vision loss.

The tear film consists of 3 layers: an inner mucous layer, a middle aqueous layer, and an oily outer layer (2). The outer layer is composed of meibum, a substance that contains a combination of various polar and nonpolar lipids and proteins (3, 4) and is produced by the Meibomian glands. Meibomian glands are holocrine sebaceous glands located in the tarsal plate of both the upper and lower eyelids. They consist of several acini organized around a central duct; the acini contain epithelial cells called meibocytes that secrete meibum; after meibocytes mature and terminally differentiate, they rupture to release their lipid content (4, 5). Thus, the meibocyte population is constantly renewed/replenished throughout life, and there must exist an adult stem cell population that regulates this homeostasis (6). Meibum secreted from the Meibomian glands functions to maintain moisture on the ocular surface by protecting the aqueous layer from evaporation, while also protecting the eye from environmental threats and infections (7, 8). Hence, when meibum production is compromised by Meibomian gland dysfunction, the tear film is destabilized, resulting in evaporative dry eye disease, the most common type of dry eye disease (9).

In the mouse, Meibomian gland morphogenesis is initiated by the formation of a placode in the epithelial layer of the eyelid embryonically at E18.5. This is followed by the invagination of the epithelium into the adjacent mesenchyme on P1–P3. Subsequently, the invaginated epithelium progressively elongates into the eyelid, and on P8, the meibocyte stem cells begin to differentiate to complete the full formation of the Meibomian gland by P15 (10, 11). In addition to showing a similar pattern between mice and humans, Meibomian gland morphogenesis also resembles hair follicle development. While morphological development of Meibomian glands has been well described (12), the identity of the cells of origin that give rise to the Meibomian gland during development as well as during Meibomian gland homoeostasis are less well defined.

Meibomian gland dysfunction encompasses a variety of conditions characterized by a defect in Meibomian gland function and accounts for approximately 87% of dry eye disease cases (9, 13). The clinical manifestations of Meibomian gland dysfunction include eye irritation and distress and traces of ocular inflammation (13, 14). Although the underlying molecular mechanisms are not well known, Meibomian gland dysfunction is reported to mostly occur from reduced meibum secretion, resulting from abnormal Meibomian gland activity, blockage of the Meibomian gland orifice, or structural defects such as Meibomian gland atrophy or dropout (i.e., loss of Meibomian gland). The risk factors of Meibomian gland dysfunction include aging, wearing of contact lenses, environmental challenges, drug treatments, and congenital disorders (14). One suggested cause underlying Meibomian gland structural defect is stem cell exhaustion, in which Meibomian gland–resident stem cells are unable to replenish meibum-producing meibocytes, resulting in Meibomian gland dropout (5, 14, 15). Thus, a better understanding of the identity of the cells underlying Meibomian gland development and homeostasis is key to identifying potential therapies for dry eye disease.

KROX20, also known as EGR2, is a zinc finger transcription factor that plays a role in a number of developmental processes, including Schwann cell myelination (16), hindbrain segmentation (17), lymphocyte immune responses (18), and adipocyte differentiation (19, 20). Traditionally, KROX20-expressing cells were believed to solely emerge from the neural crest during development. However, a recent study from our laboratory reported KROX20 as a marker for a newly identified stem cell population from epithelial lineage (21). These KROX20-positive cells differentiate into hair shaft progenitor cells in the hair matrix and ultimately constitute the structural component of the hair shaft. In the hair matrix, *Krox20* lineage cells also induce melanocyte differentiation to generate hair pigmentation (21).

During our characterization of mice lacking KROX20 protein in epithelial cells, we serendipitously observed that these mice developed squamous metaplasia of the ocular surface, a hallmark of dry eye disease (22, 23). Histological analyses revealed that these mice lacked Meibomian glands. Here, we report the identification of *Krox20* as an important driver of Meibomian gland development and homeostasis as well as potentially novel models for studying dry eye disease secondary to Meibomian gland dysfunction.

## Results

### Deletion of KROX20 in epithelial cells results in corneal hyperkeratinization characteristic of dry eye disease.

**Figure 1:**
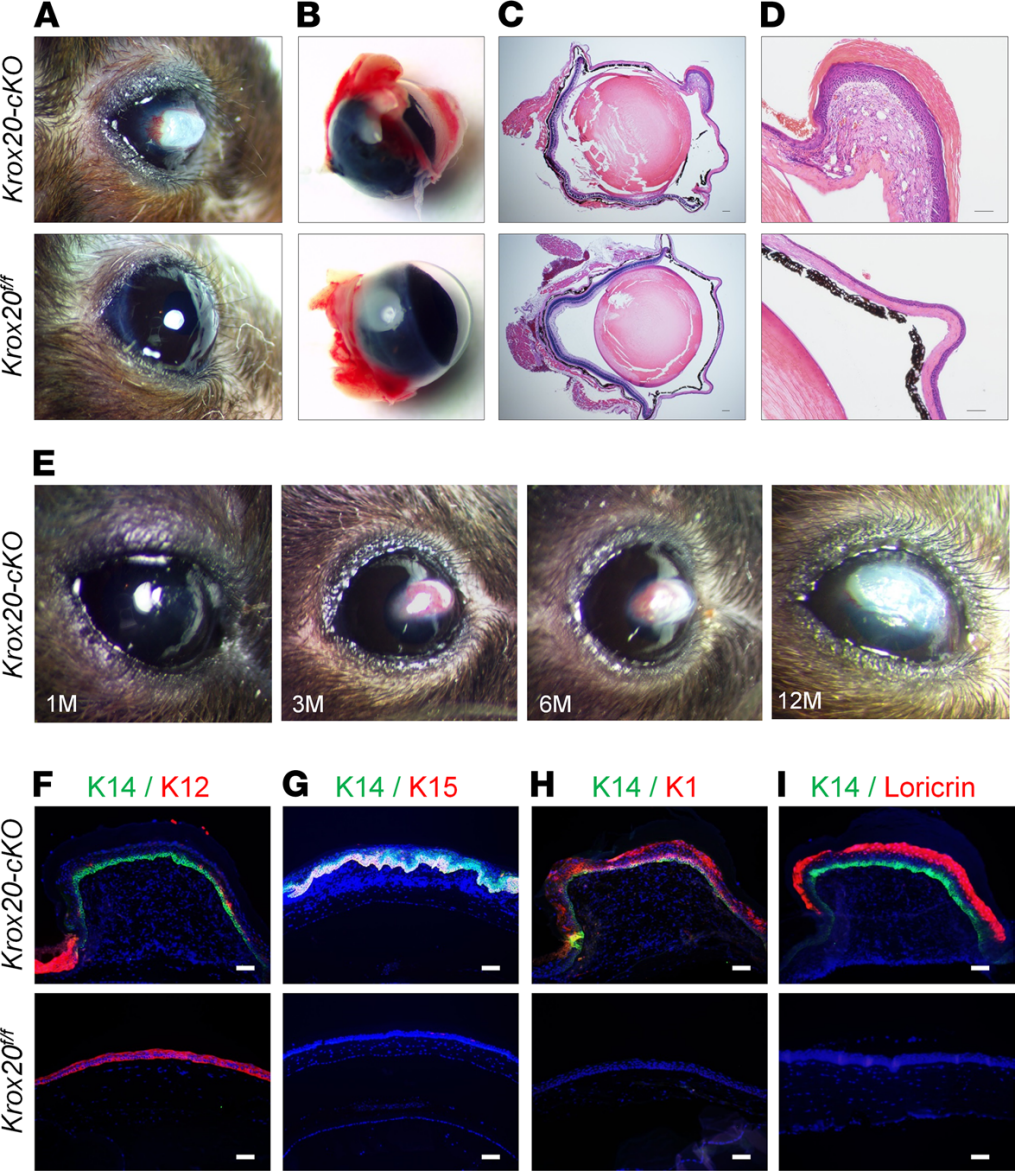
Deletion of KROX20 protein in epithelial lineage cells results in dry eye–mediated squamous metaplasia of the cornea. (**A**–**D**) *Krox20^fl/fl^;K14-Cre* *(Krox20-cKO)* mice exhibit hyperkeratinization of the cornea. (**A** and **B**) Gross images of the eye showing the corneal lesions in mutant mice (*Krox20-cKO*) compared with control mice (*Krox20^fl/fl^*). (**C** and **D**) H&E staining of a section of an eye showing a normal corneal surface in *Krox20^fl/fl^* controls and squamous metaplasia in the *Krox20* mutant mice. (**E**) *Krox20-cKO* mice show a worsening corneal phenotype as they age (*n* = 6). (**F**–**I**) Coimmunostaining of K14 with (**F**) corneal epithelium marker K12 and (**G***–***I**) stratified epidermal markers (**G**) K15, (**H**) K1, and (**I**) loricrin in *Krox20-cKO* mice and *Krox20^fl/fl^* littermate controls. *n* = 26 *Krox20^fl/fl^* mice, and *n* = 39 *Krox20-cKO* mice. Among mice analyzed, 100% of *Krox20-cKO* mice developed corneal lesions. Representative images are shown. M, months. Scale bar: 100 μm.

We previously identified KROX20 as a marker of a novel stem cell population that gives rise to hair (21). To further investigate the role of the KROX20 protein in epithelial cells, we generated *Krox20^fl/fl^*;*K14-Cre* (*Krox20-cKO*) mice. Unexpectedly, we found that between 1 and 3 months of age, *Krox20-cKO* mice began to develop squamous metaplasia of the ocular surface, a classic hallmark of dry eye (Figure 1, A–E). Whereas WT littermates maintained a normal ocular surface, with smooth and transparent corneas, the scabrous ocular surface in *Krox20-cKO* mice progressively worsened as they aged (Figure 1E). Notably, corneal lesions were observed in 100% of *Krox20-cKO* mice. H&E analysis revealed structural distortion of the cornea, along with inflammation, neovascularization, and hyperkeratinization (Figure 1, C and D). The squamous metaplasia observed in dry eye is generally characterized by abnormal differentiation of the corneal epithelium, in which corneal-specific keratins are replaced by epidermal keratins. Immunofluorescence staining revealed complete absence of the corneal epithelial marker K12 in the scabrous area of the cornea (Figure 1F), while stratified epidermal markers (K15, K1, and loricrin) were present (Figure 1, G–I). These results indicate that elimination of KROX20 expression in the K14 lineage results in squamous metaplasia of the cornea, a manifestation of severe dry eye.

### KROX20 expression is required for Meibomian gland development/morphogenesis and function.

**Figure 2:**
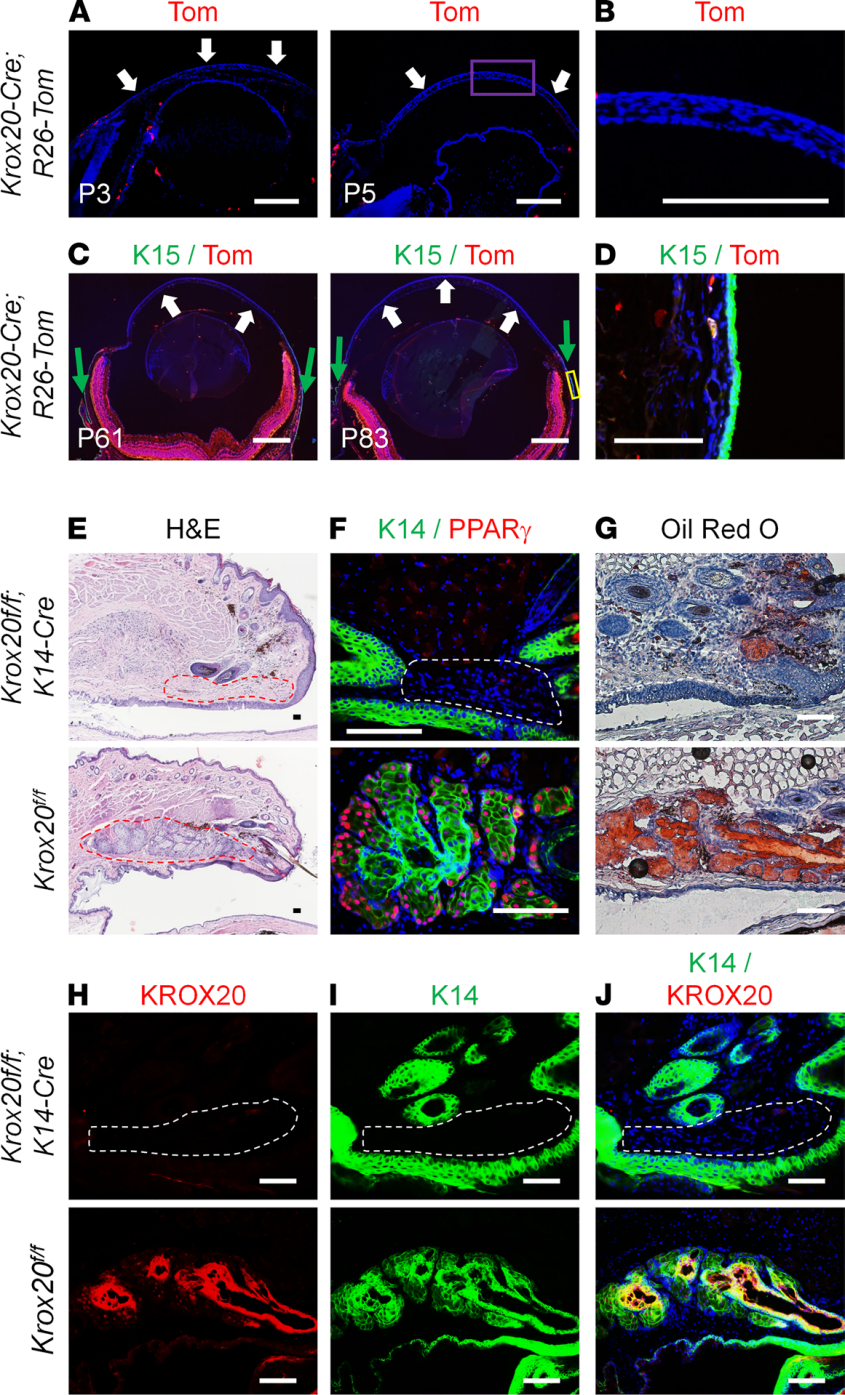
KROX20 protein is required for Meibomian gland development. (**A** and** C**) Lineage tracing in *Krox20-Cre; R26-tdTomato* (*Krox20-Cre; R26-Tom*) mice shows absence of KROX20 expression or *Krox20*-lineage cells in the cornea (white arrows) and the limbus (green arrows). (**B**) The purple boxed region in **A**, which displays an image of “P5 eye,” is shown, with a high-magnification view of the cornea. (**C**) Immunofluorescence staining of eyeballs from *Krox20-Cre;R26-Tom* mice for limbus marker K15 and tdTomato (Tom). (**D**) The yellow boxed region in **C**, which displays an image of “P83 eye,” is shown, with a high-magnification view of the limbus. (**E**) H&E staining of 2.5-month-old *Krox20-cKO* mice shows absence of the Meibomian gland. (**F**) Immunofluorescence staining for K14 costained with PPARγ. (**G**) Oil Red O staining showing absence of meibum in *Krox20-cKO* mice. (**H**–**J**) Immunofluorescence staining of Meibomian gland with KROX20 and K14 antibodies. Dashed lines (red in **E** and white in **G**–**J**) demarcate the expected location of the Meibomian gland. *n* = 20 *Krox20^fl/fl^* mice, and *n* = 25 *Krox20-cKO* mice. Among mice analyzed, 100% of *Krox20-cKO* mice lack the Meibomian gland. Representative images are shown. Scale bar: 100 μm.

The corneal defects that we observed in the *Krox20-cKO* mice could either be intrinsic to the corneal epithelium or extrinsic, e.g., due to a defect in the Meibomian gland. We therefore crossed *Krox20-Cre* mice (24) to a lineage-tracing reporter, *R26-TdTomato*, to generate *Krox20-Cre; R26-Tom* mice, and looked to see if *Krox20* was expressed in the cornea and/or the limbus, the junction between the cornea and the sclera, using K15 as a marker of the limbus (25). The lack of KROX20-expressing cells or *Krox20*-lineage cells in the cornea or in the limbus showed that not only is KROX20 not expressed in these tissues, but *Krox20*-lineage cells don’t differentiate to generate the tissues. (Figure 2, A–D). This indicated that the corneal phenotype we observed in *Krox20-cKO* mice was due to extrinsic factors and could possibly be a secondary effect due to Meibomian gland malfunction or defect, because hyperkeratinization of the cornea can result from Meibomian gland dysfunction (9, 13). We therefore harvested the eyelids of these mice for analysis of the Meibomian glands. H&E staining of the eyelids revealed a complete absence of Meibomian gland structure in all *Krox20-cKO* mice analyzed (Figure 2E), which was confirmed by lack of expression of 2 Meibomian gland markers: K14, a keratin that is expressed in both the acinar and ductal region of the Meibomian gland (26), and PPARγ, a marker of meibocytes (10) (Figure 2F). (The K14 staining observed in this image is from the hair follicle.) As there was no visible Meibomian gland, the *Krox20-cKO* mice failed to produce meibum, as shown by the lack of Oil Red O staining (Figure 2G), which stains neutral triglycerides and lipids. Immunohistochemistry with KROX20 antibody confirmed the absence of KROX20 protein in the eyelids of *Krox20-cKO* mice (Figure 2, H–J). These data demonstrate that epithelial KROX20 expression is essential for Meibomian gland development and/or maintenance.

### KROX20 is expressed in the developing and adult Meibomian gland, and Krox20-lineage cells give rise to the Meibomian gland.

**Figure 3:**
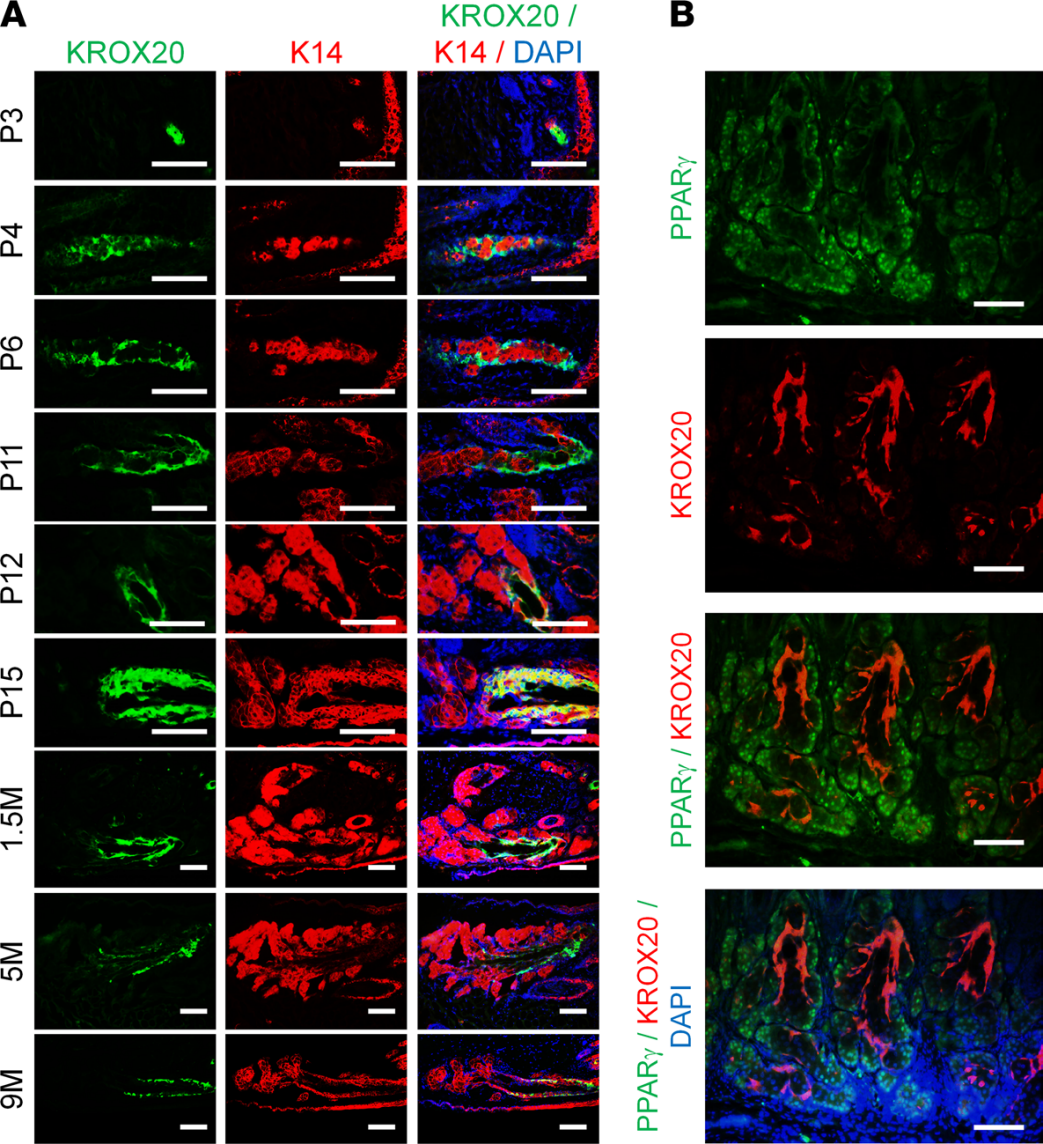
KROX20 is expressed in Meibomian gland during development and maintained throughout life. (**A**) Immunofluorescence staining of *Krox20-GFP* mice demonstrates KROX20 expression in the Meibomian gland at various ages. (**B**) Immunofluorescence staining for GFP (KROX20) and PPARγ expression in Meibomian gland of P12 mice. Representative images are shown. M, months. Scale bar: 100 μm.

To determine the KROX20 expression pattern in the Meibomian gland, we took advantage of a *Krox20-GFP* knockin mouse line (27) that allowed us to monitor *Krox20* expression with a GFP reporter. Because this reporter line is a knockin and not a transgenic, GFP expression faithfully recapitulates endogenous KROX20 expression. KROX20 expression was detected in the epithelial cord during the early stages of Meibomian gland morphogenesis prior to the formation of the acini (P3–P6) (Figure 3A) and persisted throughout life. However, as the branching of the acini became apparent (>P11), we observed that KROX20-positive cells were selectively concentrated in the suprabasal layer of the ductal region (Figure 3A) and distinct from PPARγ-expressing cells, which are limited to the acinar region at P12 (Figure 3B).

**Figure 4:**
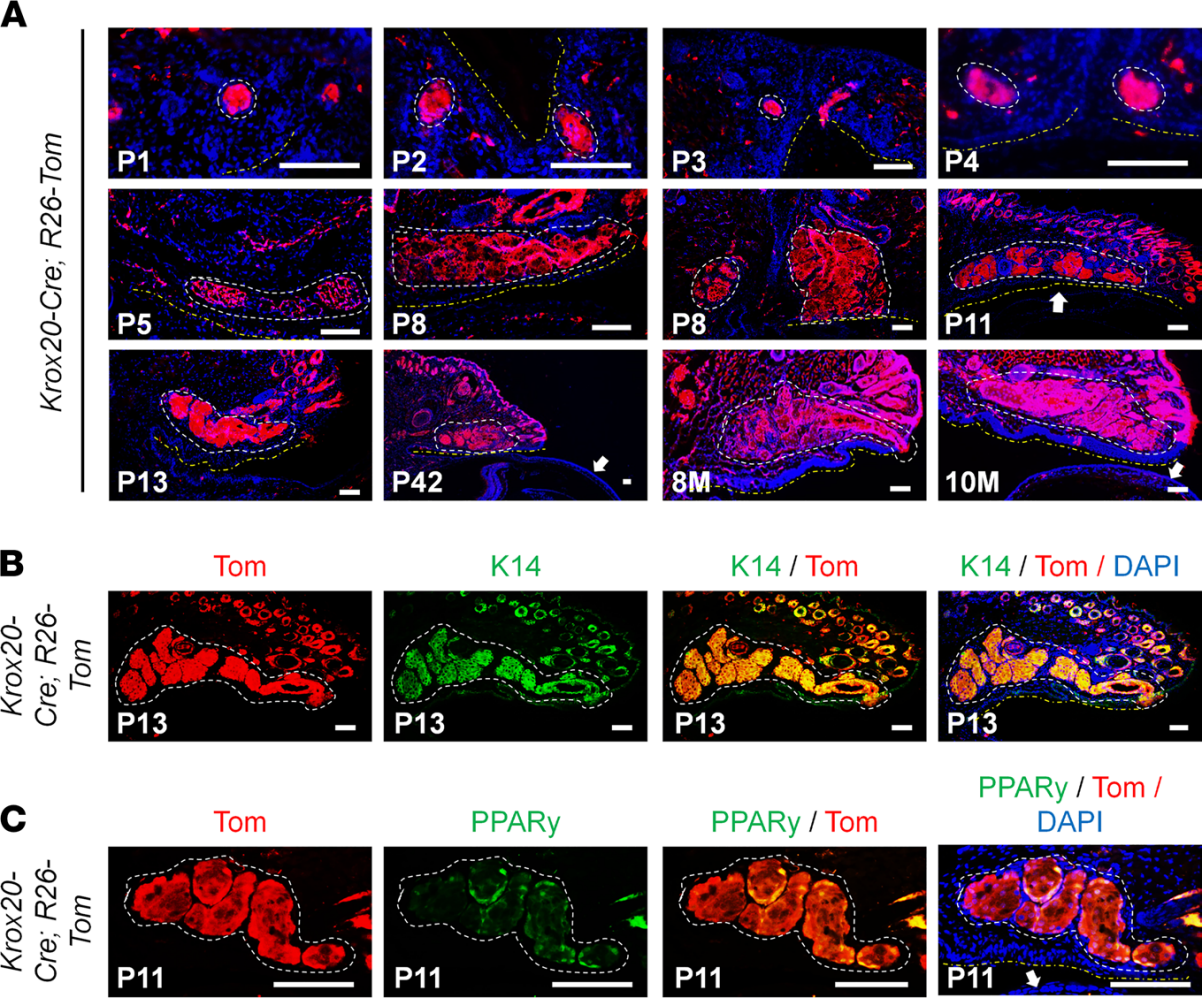
*Krox20*-lineage tracing in the Meibomian gland. (**A**) Lineage tracing of *Krox20* with tdTomato Red reporter (*Krox20-Cre R26-Tom*) shows the distribution of *Krox20*-lineage cells in the Meibomian gland during development (<P15) and after the Meibomian gland structure is fully established (>P15). Sections are also stained with DAPI. (**B**) Immunofluorescence staining of the Meibomian glands from *Krox20-Cre R26-Tom* mice (P13) for Meibomian gland marker K14 and tdTomato (Tom). (**C**) Immunofluorescence staining of the Meibomian gland from *Krox20-Cre R26-Tom* mice (P11) for meibocyte marker PPARγ and Tom. White dashes in **A**–**C** encircle the Meibomian gland; yellow dashes in **A** demarcate the conjunctival surface of the eyelid; and white arrows indicate the cornea. Representative images are shown. M, months. Scale bar: 100 μm.

To investigate the contribution of KROX20-expressing cells in Meibomian gland development, we used the *Krox20-Cre; R26-Tom* mice. In these mice, the expression of tdTomato marks both the cells actively expressing KROX20 and their differentiated progeny. Interestingly, tdTomato signal was detected in the entire Meibomian gland at various time points during Meibomian gland morphogenesis and development (P1–P13) and after the Meibomian gland was fully formed (P42 to 10 months [P42–10M]) (Figure 4, A and B). Meibocytes of the duct and acini begin to differentiate at P8, suggesting that prior to this time the cells contained within the Meibomian gland were mostly stem and progenitor meibocytes. As such, the presence of tdTomato signal in the P1–P8 Meibomian gland indicates these stem/progenitor meibocytes consist of *Krox20*-lineage cells. Furthermore, *Krox20*-lineage cells and/or KROX20-expressing cells generate the full, mature Meibomian gland structure, as evidenced by tdTomato signal in the entire gland. Staining for K14 showed overlap with the tdTomato signal, demonstrating that the *Krox20*-lineage cells do indeed give rise to the Meibomian gland (Figure 4B). PPARγ, a marker of undifferentiated meibocytes, is first expressed in the Meibomian gland at P3 and correlated with meibum production (10). In addition to being detected in the Meibomian gland at an earlier age (P1) (Figure 4A), *Krox20*-lineage cells also included PPARγ-expressing meibocytes (Figure 4C). These data indicate that, although KROX20 expression is limited to the ductal region, the entire Meibomian gland, including PPARγ-expressing meibocytes, is generated from *Krox20*-lineage cells, suggesting that KROX20 marks a population of stem/progenitor cells that differentiate to generate both the duct and the acini of the Meibomian gland.

### Deletion of epithelial-derived Krox20-lineage cells prevents Meibomian gland development.

**Figure 5:**
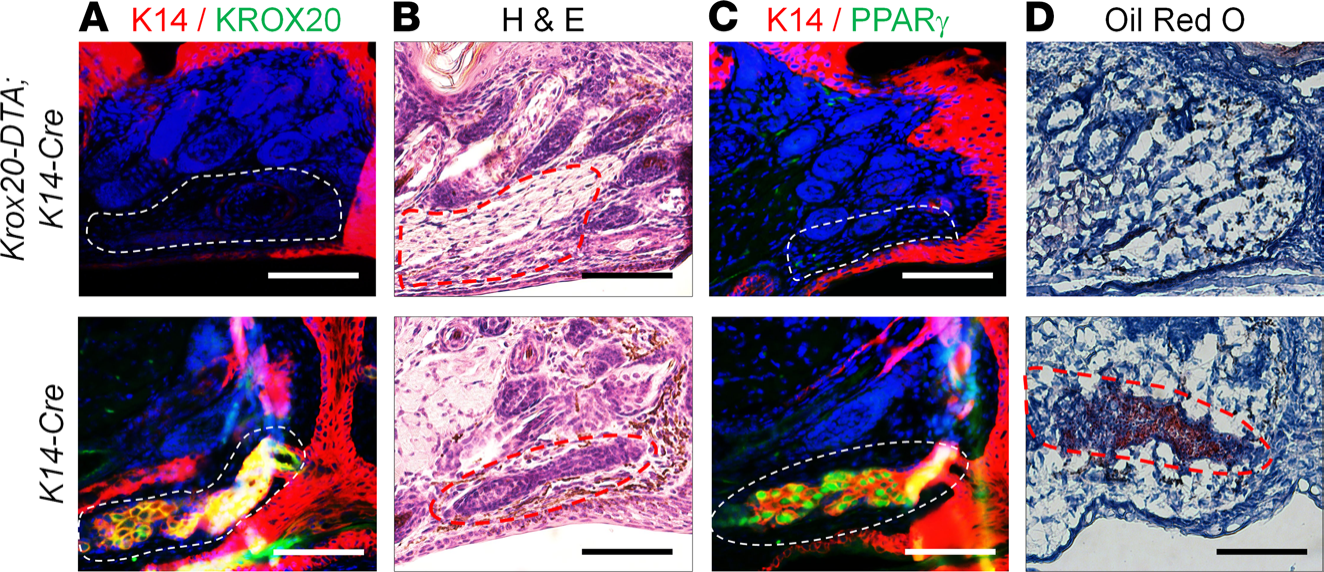
KROX20-positive cells are indispensable for Meibomian gland development. *Krox20-DTA*;*K14-Cre* pups (P6) in which KROX20-expressing cells were deleted exhibit complete loss of Meibomian gland, as shown by coimmunostaining for (**A**) K14 and KROX20, (**B**) H&E staining, and (**C**) K14 and PPARγ immunostaining. (**D**) These mice also failed to produce meibum, as shown by the lack of Oil Red O staining in the eyelid. Dashed lines (red in **B** and **D** and white in **A** and **C**) demarcate the Meibomian glands in the control mice and its expected location in the *Krox20-DTA;K14-Cre* mice. *n* = 7 *K14-Cre* mice, and *n* = 11 *Krox20-DTA;K14-Cre* mice. Representative images are shown. Scale bar: 100 μm.

The expression of KROX20 in a subset of cells during Meibomian gland development and the absence of Meibomian gland upon deletion of *Krox20* led us to examine the functional role of *Krox20*-expressing cells in the Meibomian gland. To do this, we ablated epithelial *Krox20*-lineage cells by crossing mice harboring the *Krox20-lox-Stop-lox-DTA* (*Krox20-DTA*) knockin allele (27) with a *K14-Cre* line to generate *Krox20-DTA;K14-Cre* mice. In this model, KROX20-positive cells of the K14 lineage express diphtheria toxin A (DTA) and are therefore ablated. Although perinatally these mice are indistinguishable from their littermate controls, they start showing signs of sickness between P3 and P7 and die within 24–36 hours, most likely due to defects in development of vital internal organs, limiting the age at which they can be analyzed. Nonetheless, immunostaining of the eyelids of P6 *Krox20-DTA;K14-Cre* mice showed a complete absence of KROX20-positive cells (Figure 5A), confirming that KROX20-positive cells had indeed been completely ablated by DTA. Furthermore, histological analyses revealed the absence of Meibomian glands in these mice, as evidenced by H&E staining and the lack of Meibomian gland markers K14 and PPARγ (Figure 5, B and C). Oil Red O staining, which marks lipids present in meibum, was also undetectable in the eyelids of *Krox20-DTA*;*K14-Cre* mice (Figure 5D), further demonstrating the absence of Meibomian gland.

### Deletion of Krox20-lineage cells in fully developed Meibomian glands impairs Meibomian gland homeostasis and causes corneal lesions.

**Figure 6:**
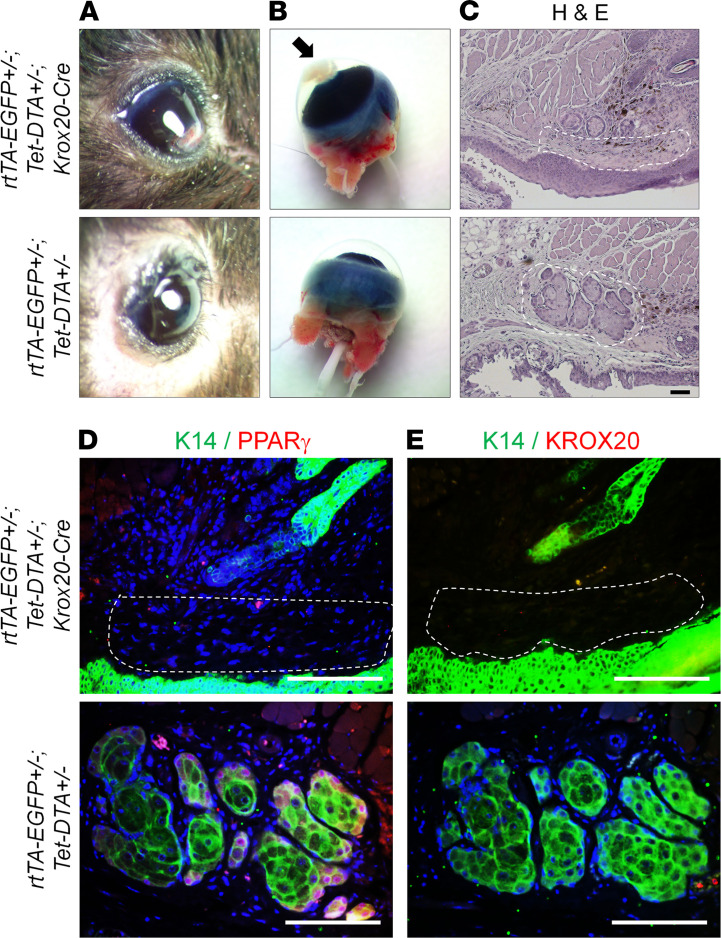
Inducible ablation of *Krox20*-lineage cells in adult mice. Doxycycline-induced *Krox20*-lineage cell ablation from P20 to P92 causes corneal lesions. (**A** and **B**) Gross images of eyes and eyeballs from *rtTA-EGFP^+/–^;Tet-DTA^+/–^;Krox20-Cre* mice and controls. The black arrow in **B** is pointing to the corneal lesion. (**C**) H&E staining as well as (**D**) immunofluorescence staining for K14 and PPARγ and (**E**) K14 and KROX20 in the Meibomian glands from *rtTA-EGFP^+/–^*;*Tet-DTA^+/–^*;*Krox20-Cre* mice and controls. White dashed lines in **C**–**E** demarcate the Meibomian gland in the control mice and its expected location in the *rtTA-EGFP^+/–^*;*Tet-DTA^+/–^*;*Krox20-Cre* mice. *n* = 7 *Krox20-DTA;K14-Cre* mice, and *n* = 11 *Krox20-DTA;K14-Cre* mice. Representative images are shown. Scale bar: 100 μm.

To determine the role of *Krox20*-lineage cells in fully developed Meibomian glands, we used an inducible system — *Krox20-Cre;R26-rtTA;Tet-DTA* mice — to delete these cells in P20 mice, after the Meibomian glands are fully formed. In these mice, the reverse tetracycline-controlled transactivator (rtTA) (19) is expressed in *Krox20*-lineage cells (*Krox20-Cre;R26-rtTA*). The *tetO-DTA* expresses DTA (28) to ablate cells with rtTA expression (i.e., *Krox20*-lineage cells) upon doxycycline induction. P20 mice were treated with doxycycline for 2 months (until P94) and monitored for corneal defects. Interestingly, the doxycycline-treated mice began to develop corneal lesions (Figure 6, A and B), and histological analysis of these mice revealed the loss of Meibomian glands, as evidenced by H&E staining (Figure 6C) and immunostaining of K14, PPARγ, and KROX20 (Figure 6, D and E). These results demonstrate the importance of KROX20-expressing cells in maintaining homeostasis of the adult Meibomian gland.

### Depletion of both KROX24 and KROX20 accelerates corneal hyperkeratinization.

**Figure 7:**
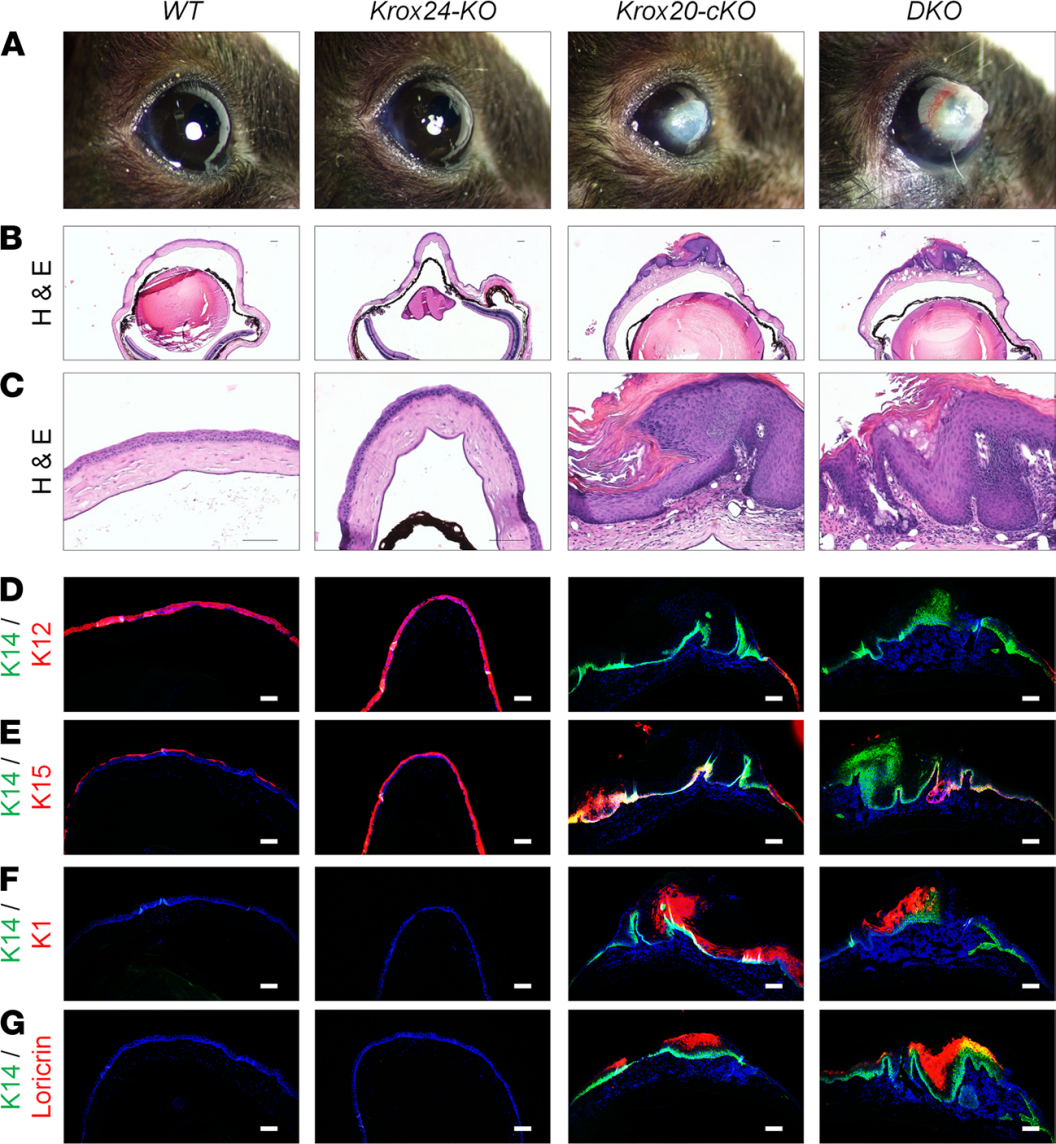
Ablation of both KROX20 and KROX24 increases severity of dry eye. (**A**) Gross images of the eye showing corneal lesions in *Krox20^fl/fl^;K14-Cre* (*Krox20-cKO*)** and *Krox24^–/–^;Krox20^fl/fl^;K14-Cre* (*DKO*) mice, while *Krox24^–/–^* (*Krox24-KO*) and *Krox20^fl/fl^* (WT) controls maintained normal ocular surface. (**B** and **C**) H&E staining of a section of an eye showing squamous metaplasia in *Krox20-cKO* and *DKO* mice compared with *Krox24-KO* mice and controls. (**D**–**G**) Immunofluorescence staining of the corneas of WT, *Krox24-KO*, *Krox20-cKO*, and *DKO* mice for (**D**) corneal epithelium marker K12 and stratified epidermal markers (**D**–**G**) K14, (**E**) K15, (**F**) K1, and (**G**) loricrin. Gross images in **A** are the same as those in Figure 8A, Supplemental Figure 1 (8M), and Supplemental Figure 2A. *n* = 50 mice for each genotype. Among mice analyzed, 100% of *Krox20-cKO* and *DKO* mice developed corneal lesions. Representative images are shown. Scale bar: 100 μm.

KROX20 is a member of the early growth response (EGR1–EGR4) family of zinc finger transcription factors (29). KROX20 and KROX24 (EGR1) have been reported to have overlapping, distinct, and opposing functions in various scenarios (30–34). Interestingly, in T lymphocytes, ablation of both *Krox20* and *Krox24* in vivo resulted in a synergistic effect compared with loss of either of them alone (34). Therefore, to investigate this potential dynamic in Meibomian gland development and function, we compared the eye phenotypes of *Krox24^–/–^* (*Krox24-KO*) (35), *Krox20-cKO*, *Krox24^–/–^*;*Krox20^fl/fl^*;*K14-Cre* (*DKO*), and WT littermates (Figure 7). As expected, *Krox20-cKO* mice developed dry eye, which was characterized by a scabrous ocular surface and hyperkeratinization (Figure 7). However, *Krox24-KO* mice were identical to WT littermate controls, maintaining a smooth and normal ocular surface (Figure 7, A–C). Similar to *Krox20-cKO* mice, the *DKO* mice also developed squamous metaplasia, as evidenced by the presence of corneal lesions, inflammation, and hyperkeratinization (Figure 7, A–C). Furthermore, corneal epithelial differentiation was disrupted, resulting in replacement of K12-expressing corneal epithelium with keratinized, stratified epithelium expressing K14, K1, and loricrin (Figure 7, D–G). Interestingly, although *Krox24-KO* mice did not develop a Meibomian gland or corneal phenotype, we did observe that corneal lesions appeared earlier in the *DKO* mice compared with the *Krox20-cKO* mice and that progression of phenotype severity was considerably accelerated (Supplemental Figure 1; supplemental material available online with this article; https://doi.org/10.1172/jci.insight.151078DS1).

**Figure 8:**
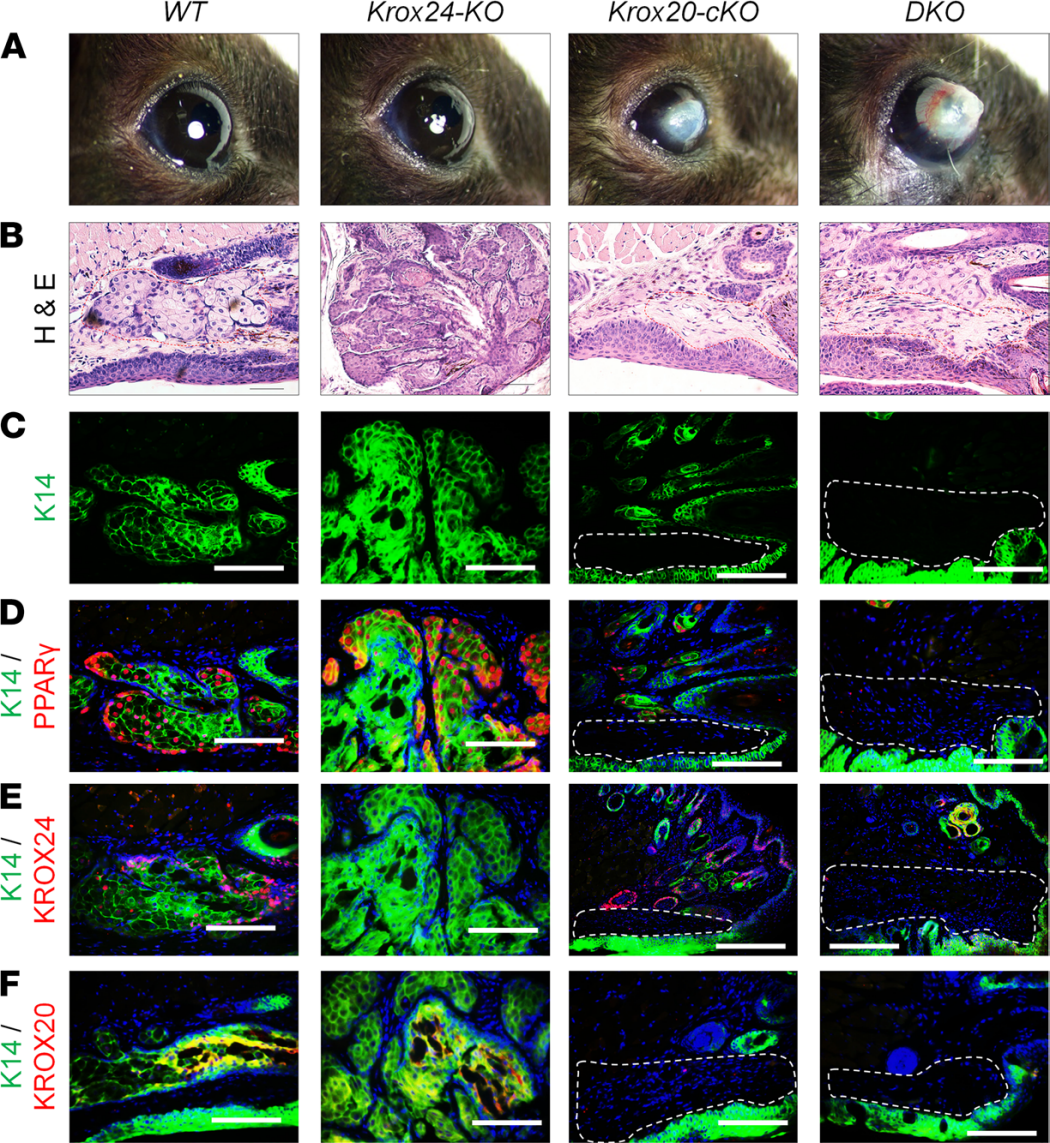
Absence of the Meibomian gland in mice lacking both *Krox20* and *Krox24*. (**A**) Gross images of the eye in *Krox20^fl/fl^* (WT), *Krox24^–/–^* (*Krox24-KO*), *Krox20^fl/fl^;K14-Cre* (*Krox20-cKO*), and *Krox24^–/–^;Krox20^fl/fl^;K14-Cre* (*DKO*) mice. (**B**–**F**) Histological analyses revealed the absence of the Meibomian gland (MG) in the eyelids of *Krox20-cKO* and *DKO* mice,** whereas *Krox24-KO* and WT** littermates maintained normal MG structure. Gross images in **A** are the same as those in Figure 7A, Supplemental Figure 1 (8M), and Supplemental Figure 2A. Dashed lines (red in **B** and white in **C**–**F**) demarcate the Meibomian gland in the control mice and its expected location of the Meibomian glands in the KOs. *n* = 20 mice for each genotype. Representative images are shown. Scale bar: 100 μm.

To examine the glandular structure associated with these corneal manifestations (Figure 8A), we performed histological analyses of the eyelids (Figure 8, B–F). As expected, the Meibomian gland was absent in the *Krox20-cKO* and *DKO* mice, while the *Krox24-KO* mice maintained normal Meibomian gland structure (Figure 8, B–F). The ability of *Krox24-KO* mice to maintain normal Meibomian gland structure and function depends on the presence of KROX20-positive cells and normal KROX20 expression (Figure 8F). Since KROX20 was not detected in the cornea, we evaluated whether the phenotype observed in the *Krox20-DKO* mice was correlated with corneal KROX24 expression. Similar to KROX20, KROX24 was also not expressed in the cornea (Supplemental Figure 2). Hence, the *Krox20-DKO* corneal defect is independent of KROX24 expression in the cornea. The combination of these results suggests that, although loss of *Krox24* alone is insufficient to cause abnormal Meibomian glands leading to corneal lesions, it acts synergistically with epithelial *Krox20* ablation to accelerate the phenotypic severity in the *DKO* mice.

## Discussion

In this study, we evaluated the role of epithelial KROX20 in Meibomian gland development and maintenance. We observed sustained KROX20 expression in Meibomian glands from initial morphogenesis to complete structural formation and on into adulthood. Of note, while KROX20 expression was mostly limited to the central duct region, *Krox20*-lineage cells were observed throughout the whole Meibomian gland. These data indicate that KROX20 labels a population of stem/progenitor cells that give rise to the Meibomian gland. In support of this, Meibomian glands failed to develop in mice lacking KROX20-positive cells. Additionally, we found that knockout of the KROX20 protein itself (*Krox20-cKO*) resulted in complete absence of Meibomian glands. Consequently, these mice failed to generate meibum, causing the development of squamous metaplasia of the cornea, a severe manifestation of dry eye disease. Of note, this corneal phenotype was observed in 100% of *Krox20-cKO* mice. In all of the many studies published using various Cre recombinase drivers, this phenotype has not been reported and is therefore not likely to be due to Cre expression or other genetic modifiers. Taken together, our findings demonstrate that not only are KROX20-positive cells essential for normal Meibomian gland development and maintenance, but expression of KROX20 in these cells is also required.

Prior to P12, KROX20-positive cells did not express K14 in the Meibomian gland (Figure 3A); however, K14-Cre effectively deleted Krox20 cells and protein, as evidenced by the severity of the phenotypes observed. This is because it has previously been established that epithelial Krox20 cells come from the embryonic K14 lineage (21), and so recombination of the loxP sites with K14-Cre likely happens very early in embryonic development. Thus, the early deletion of Krox20 by K14-Cre results in absence of the Meibomian gland. However, it is possible that, while epithelial Krox20-expressing cells are derived from K14-expressing cells during the initial embryonic stages of ectoderm-derived epithelium development (21), K14 expression in these cells can fluctuate, i.e., turn off and on, in the developing Meibomian gland. Hence, although the Meibomian gland is derived from the K14-lineage, KROX20 is expressed in cells that are no longer expressing K14 at that early time of Meibomian gland development.

When *Krox24*, a different member of the EGR family was ablated, the cornea maintained normal structure and smoothness, while the loss of both *Krox20* and *Krox24* accelerated the development and severity of the dry eye–mediated corneal lesion. This indicates that although *Krox24* ablation alone is not sufficient to disrupt the structural integrity of the Meibomian gland, it functions synergistically with *Krox20* loss to accelerate and exacerbate the phenotype, suggesting that KROX20 expression is able to fully compensate for the loss of *Krox24*, while *Krox24* is not sufficient to sustain tissue integrity in the absence of *Krox20*.

Several reports have described the generation of animal models to mimic the physiological features observed in human Meibomian gland dysfunction–mediated dry eye disease (reviewed in refs. 9, 36). In some of these models, Meibomian gland function was altered through mechanical interventions or chemical treatments. In other cases, abnormal Meibomian glands were observed in a variety of transgenic and genetically mutant mice (9, 36, 37). However, for the most part, these studies focused on analyzing the structural and functional abnormalities of Meibomian glands, while the corresponding effects on the ocular surface were not well established. One study that addresses both aspects is the X-linked anhidrotic-hypohidrotic ectodermal dysplasia mouse (Tabby), harboring an ectodysplasin A (EDA) mutation. In this mouse model, the Meibomian gland is completely absent, resulting in several defects in the ocular surface, including keratitis, corneal ulceration, neovascularization, keratinization, blepharitis, and conjunctivitis (23, 38). In another example, conditional deletion of *Notch1* with *K14-Cre* led to the development of abnormal Meibomian glands and a corneal phenotype (37). Notably, these results are similar to the phenotype observed in our *Krox20-cKO* mice, providing a platform to evaluate the relationship in the mechanisms underlying the pathophysiology represented between these different mouse models.

Meibomian gland dysfunction accounts for the majority of reported dry eye disease cases, and there is an urgent need to develop effective therapies for this condition. Unfortunately, while the morphological development of Meibomian glands is well described, the cell dynamics underlying Meibomian gland dysfunction–mediated dry eye disease remain poorly elucidated (9). In addition to providing a robust mouse model for Meibomian gland dysfunction impact on the ocular surface and for therapeutic screening, the identification of a stem cell population that gives rise to the Meibomian gland would overcome a significant barrier, allowing for a deeper and more precise understanding of the intricacies of Meibomian gland development, differentiation, and homeostasis.

## Methods

### Mice.

All experiments in this study were performed at the University of Texas Southwestern Medical Center. To ensure the welfare of the mice, mice were monitored daily and sacrificed as soon as any sign of sickness was observed. For mice that developed the eye lesion phenotype, we, along with the ARC veterinary technicians, monitored the mice daily, to ensure that the overall health/lifestyle (e.g., eating, drinking, activity, etc.) of the mice was not negatively affected. In addition, eye ointment was regularly applied to relieve the dry eye symptoms. Both male and female mice were used for each experiment. *Krox24-KO* mice (35), *K14-Cre* mice (39), *R26-rtTA* mice (19), and *tetO-DTA* mice (28) were purchased from The Jackson Laboratory. The *Krox20-Cre* mouse line is described in Voiculescu et al. (24), and the *Krox20^fl/fl^* mouse line is described in Taillebourg et al. (40). *Krox20-lox-GFP-lox-DTA* (27) is a knockin allele. GFP serves as a reporter for the *Krox20* promoter in the absence of Cre; however, in the presence of Cre, GFP is floxed out and DTA is turned on to ablate the cells (Supplemental Figure 3). Due to the nature of these mouse crosses, the genetic background of these mice is very mixed and includes C57BL/6, BALB/c, etc. Genotyping was performed by isolating genomic DNA from the tail of each mouse followed by PCR amplification of the specific genes of interest. Primer sequences are provided in Supplemental Table 1.

### Preparation of tissue samples for histological analysis.

For histological analysis of the cornea, mice were euthanized with CO_2_, and entire eyeballs were isolated. However, to study the Meibomian gland, each eyeball was isolated together with both the top and bottom eyelids, while maintaining their normal structure.

For paraffin sections, the tissues were immediately fixed in 10% formalin overnight and processed in ethanol, xylene, and wax, using the Citadel 2000 Tissue Processor (Thermo Fisher Scientific). Subsequently, the samples were embedded in paraffin and sectioned at 5–8 μm thickness. The tissue sections were mounted on microscope slides maintained at room temperature for subsequent immunostaining and H&E staining. H&E staining was performed following the manufacturer’s protocol (StatLab).

For frozen sections, tissues were briefly fixed in 4% paraformaldehyde for 5 minutes and incubated in OCT compound at 4°C for more than 3 hours. The samples were immediately embedded and frozen in OCT, followed by sectioning at 5 μm thickness. The tissue sections were mounted on microscope slides and maintained at less than 20°C for subsequent staining.

### Immunostaining.

For immunofluorescence staining, paraffin sections were processed following deparaffinization, rehydration, and antigen retrieval. Frozen sections were dried at room temperature and fixed in 4% paraformaldehyde for 5–10 minutes prior to the immunostaining. The tissue sections were then blocked in 10% donkey serum and immediately incubated in primary antibodies at 4°C overnight. Subsequently, the tissue samples were incubated in secondary antibody for 2 hours at room temperature and mounted with a coverslip for imaging. The primary antibodies used in this study were K1 (BioLegend, 905601), K14 (Fisher, NBP234675B), K12 (Abcam, ab185627), K15 (Abcam, ab52816), KROX20 (Invitrogen, PA5-27814), Loricrin (BioLegend, 905101), PPARγ (Cell Signaling, 2435S), and KROX24/EGR1 (Cell Signaling, 4153S). For immunofluorescence staining, the primary antibodies were detected by secondary antibodies or streptavidin conjugated with Cy3 or Alexa Fluor 488 (Jackson ImmunoResearch), and nuclei were counterstained with DAPI (Vector Labs). To verify the specificity of the primary antibodies, negative controls were used, including the tissue sections incubated without primary antibody.

### Evaluation of meibum secretion.

To evaluate meibum secretion, frozen eyeball or eyelid sections were fixed in 4% paraformaldehyde for 10 minutes, washed in PBS for 10 minutes, and stained with Oil Red O (StatLab, KTORO) following the manufacturer’s instructions. In brief, the sections were immersed in propylene glycol for 2 minutes and stained with a preheated (60°C) solution of Oil Red O for 6 minutes. Subsequently, sections were washed with 85% propylene glycol for 1 minute and rinsed with running water. Counterstaining was performed with Modified Mayer’s Hematoxylin (StatLab, KTORO).

### Microscopy.

Following the completion of immunostainings, fluorescence microscopy images were taken on an Olympus fluorescence microscope (model IX73) with software CellSens Standard (version 1.8). Error bars were added to the images using the CellSens software, and images were processed with Adobe Photoshop CS6 (version 13.0.1, ×32); procedures were limited in overall brightness/contrast adjustment and multicolor channel overlay.

### Statistics.

Statistics were performed by GraphPad Prism 8.

### Study approval.

Mouse experiments and husbandry were approved by the Institutional Animal Care and Use Committee at University of Texas Southwestern Medical Center.

## Author contributions

ET, CPL, EG, TS, and YW performed experiments. ET, CPL, and LQL analyzed the data. ET, RMM, and LQL designed the figures. ET, CPL, RMM, and LQL designed the research and contributed to writing and editing the manuscript. LQL provided supervision and acquired funding.

## Supplementary Material

Supplemental data

## Version 1 09/09/2021

In-Press Preview

## Version 2 10/22/2021

Electronic publication
