# Supplementary material for: Epithelial stem cell homeostasis in Meibomian gland development, dysfunction, and dry eye disease
Source: JCI Insight. 2021 Oct 22;6(20):e151078. doi: 10.1172/jci.insight.151078 (PMC8564894; doi:10.1172/jci.insight.151078)
Supplement: Supplemental data [file jciinsight-6-151078-s109.pdf]

Supplemental Figures and Table for:

Epithelial stem cell homeostasis in Meibomian gland development, dysfunction, and dry eye disease

Edem Tchegnon[1,2,+], Chung-Ping Liao[1,5,+,#], Elnaz Ghotbi[1] , Tracey Shipman[1], Yong Wang[1], Renee M. McKay[1], and Lu Q. Le[1,2,3,4,#]

[1]Department of Dermatology, [2]Genetics, Development and Disease Graduate Program, [3]Hamon Center for Regenerative Science and Medicine, [4]Simmons Comprehensive Cancer Center, University of Texas Southwestern Medical Center, Dallas, TX 75390, USA. [5]Graduate Institute of Medical Sciences, College of Medicine, Taipei Medical University, Taipei 110, Taiwan

[+] Authors contributed equally

Running title: Developmental origin of the Meibomian Gland

Keywords: Meibomian gland, Krox20, Dry Eye Disease, Meibomian gland dysfunction, Meibomian gland stem cells, Meibomian gland progenitor cells, Meibomian gland homeostasis, corneal lesions

Conflict of interest: The authors have declared that no conflict of interest exists.

Supplemental Figure 1

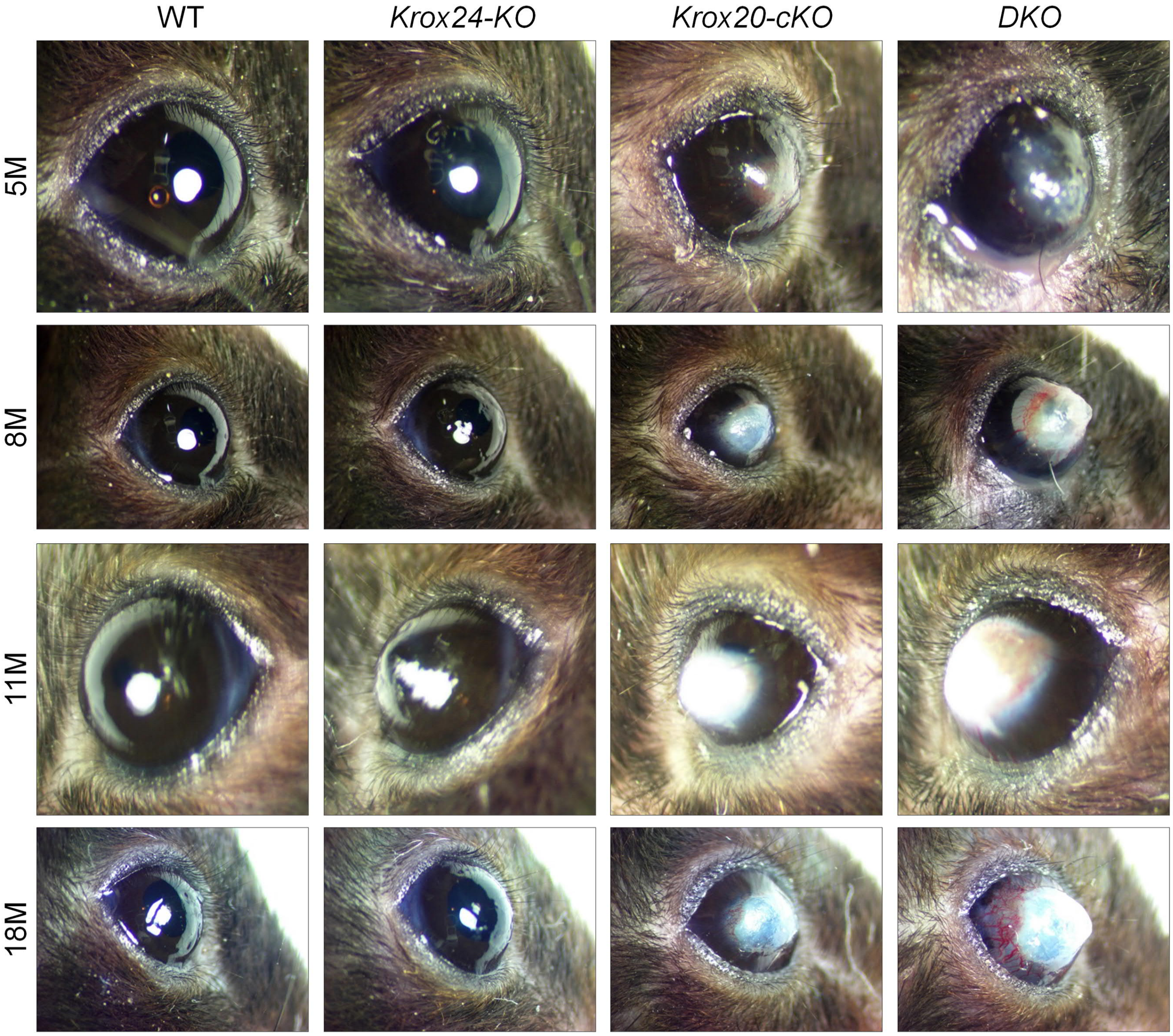

Supplemental Figure 1. Loss of both *Krox20* and *Krox24* accelerates development and progression of corneal lesion. Gross images of the eye in wild type (*WT; Krox20*$^{f/f}$), *Krox24-KO(Krox24*$^{-/-}$*), Krox20-cKO (Krox20*$^{f/f}$*;K14-Cre)*, and *DKO (Krox24*$^{-/-}$*; Krox20*$^{f/f}$*;K14-Cre)* mice at various ages (5, 8, 11, and 18 months), showing the progression of corneal lesion. Gross images shown for 8-month-old mice (8 M) are the same as in Figure 7 (panel **A**), Figure 8 (panel **A**), and Supplemental Figure 2 (panel **A**). n = 50 mice for each genotype. Among mice analyzed, 100% of *Krox20-cKO* and *DKO* mice developed corneal lesions. Representative images are shown. Scale bar represents 100 µm.

Supplemental Figure 2

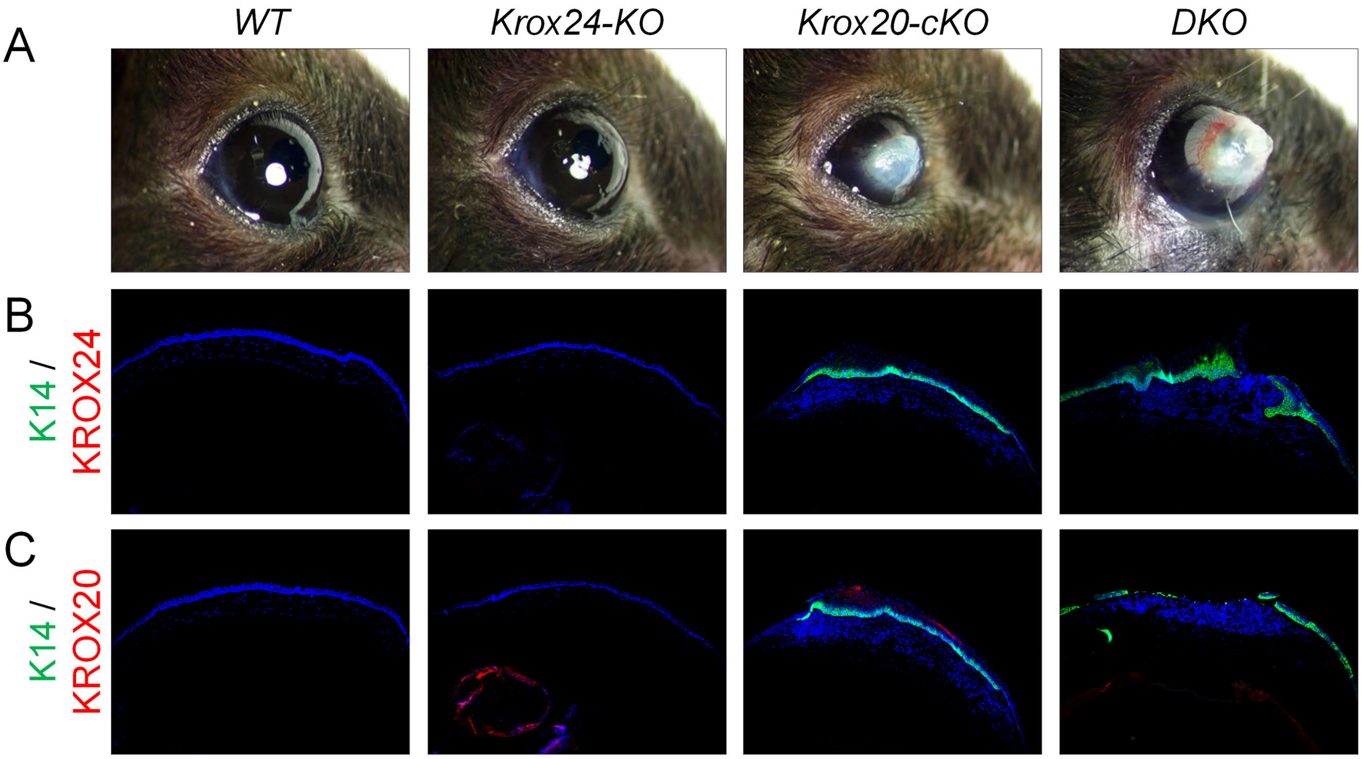

Supplemental Figure 2. Acceleration of phenotype severity is independent of KROX20 and KROX24 expression in the cornea. (A) Gross images of the eye in wild type (*WT; Krox20*$^{f/f}$), *Krox24-KO (Krox24*$^{-/-}$*), Krox20-cKO (Krox20*$^{f/f}$*;K14-Cre),* and *DKO (Krox24*$^{-/-}$*; Krox20*$^{f/f}$*;K14-Cre)* mice. Immunofluorescence staining for (**B**) K14 and KROX24, and (**C**) K14 and KROX20 in the indicated genotypes. n = 50 mice for each genotype. Among mice analyzed, 100% of *Krox20-cKO* and *DKO* mice developed corneal lesions. Representative images are shown. Gross images in (**A**) are the same as in Figure 7 (panel **A**), Figure 8 (panel **A**), and Supplemental Figure 1 (8M).

Supplemental Figure 3

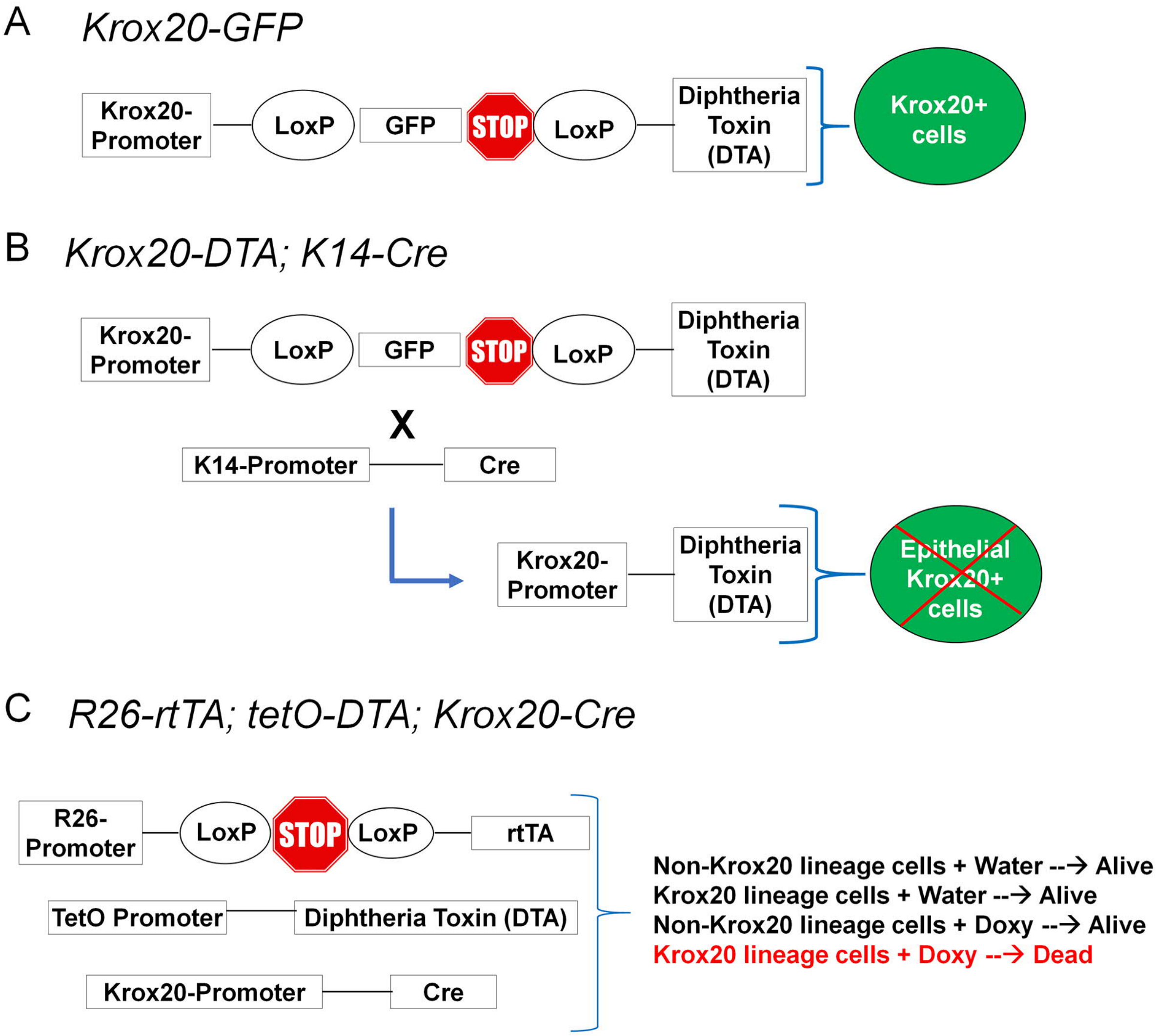

Supplemental Figure 3. Schematic illustration of Krox20 cell depletion. (A) Illustration of *Krox20-GFP* (*Krox20-Flox-GFP-Flox-DTA*). In the absence of Cre expression, GFP serves as a reporter for *Krox20* expression. However, **(B)** when crossed with *K14-Cre* to generate *Krox20-DTA; K14-Cre*, the diphtheria toxin A (DTA) is expressed, resulting in the ablation of epithelial *Krox20*-expressing cells from K14 lineage. **(C)** Illustration of *R26-rtTA; tetO-DTA; Krox20-Cre+*. When Cre is expressed under the *Krox20* promoter, it results in the DTA-mediated death of *Krox20* lineage cells upon doxycycline (doxy) treatment.

Genotype	Primer Sequence	Size
Cre	**Fwd:** CAC CCT GTT ACG TAT AGC CG<br>**Rev:** GAG TCA TCC TTA GCG CCG TA	Cre: 300bp
Beta-Actin	**Fwd:** CCT AGG CAC CAG GGT GTG AT<br>**Rev:** TCA CGG TTG GCC TTA GGG TT	ActB: 239bp
GFP	**Fwd:** GAG CTG GAC GGC GAC GTA AAC<br>**Rev:** CGT TGT GGC TGT TGT TAG TTG TAC	eGFP: 400bp
R26-TdTomato	**Fwd (Tom):** CTG TTC CTG TAC GGC ATG G<br>**Rev (Tom):** GGC ATT AAA GCA GCG TAT CC<br>**Fwd (WT):** AAG GGA GCT GCA GTG GAG TA<br>**Rev (WT):** CCG AAA ATC TGT GGG AAG TC	WT: 300bp<br>Tomato: 200bp
Krox20 fl/fl	**Fwd (WT):** GGG CTT GCA TTC TAC AGT GGT GGT C<br>**Rev (Krox20-ΔFlox):** AGT TGA CAG CCC GAG TCC AGT GG<br>**Fwd (WT; Krox20-ΔFlox):** GTG TCG CGC GTC AGC ATG CGT G<br>**Rev (WT):** GGG AGC GAA GCT ACT CGG ATA CGG	Krox20-Flox: 195bp<br>Krox20-ΔFlox: 210bp<br>WT: 162bp
Egr1 -/-	**Fwd (WT):** AAC CGG CCC AGC AAG ACA CC<br>**Fwd (KO):** CTC GTG CTT TAC GGT ATC GC<br>**Rev (Common):** GGG CAC AGG GGA TGG GAA TG	WT: 414bp<br>KO: 470bp
K14-Cre	**Fwd:** TTC CTC AGG AGT GTC TTC GC<br>**Rev:** GTC CAT GTC CTT CCT GAA GC	K14-Cre: 494 bp
Krox20-Cre	**Fwd (WT):** CGC TTC CTC GTG CTT TAC GGT AT<br>**Rev (WT):** TCA TCA GTC GGG TTA GAG CTG<br>**Rev (Mut):** GGG CTG AGG AAG ACG ACT TTA	Krox20-Cre: 480bp<br>WT: 312bp
TetO-DTA	**Fwd:** GGC GTG TAC GGT GGG AGG<br>**Rev:** GGC ATT ATC CAC TTT TAG TGC	TetO-DTA: 420bp
**Supplemental Table 1. Genotyping Primer Sequences**		
